# Periostin-Induced Wnt10a Activation Promotes Dental Pulp Stem Cell Migration During Pulp Regeneration

**DOI:** 10.3390/life15111732

**Published:** 2025-11-12

**Authors:** Keisuke Nakamura, Natsuki Iida, Yuki Hayashi, Taku Futenma, Shintaro Sakatoku, Yoshihiko Sugita, Hiroyuki Nawa

**Affiliations:** 1 Department of Pediatric Dentistry, School of Dentistry, Aichi Gakuin University, 2-11 Suemori-dori, Chikusa-ku, Nagoya 464-8651, Aichi, Japan; ag223d13@dpc.agu.ac.jp (K.N.);; 2 Department of Oral Pathology, School of Dentistry, Aichi Gakuin University, 1-100 Kusumotomachi, Chikusa-ku, Nagoya 464-8650, Aichi, Japan

**Keywords:** pulp regeneration, migration, wnt10a, periostin

## Abstract

Background: Periostin can be considered a stimulator of Wnt. Elucidating the relationship between Wnt10a and Periostin in dental pulp stem cells is considered necessary for a deeper understanding of the mechanisms of dental pulp regeneration. Methods: Regenerated dental pulp from ectopic root grafts was double-stained with BrdU and Wnt10a, and the positivity rates were analyzed. Furthermore, the expression levels of Wnt10a, LRP5/6, DKK1, and Periostin within the regenerated tissue were analyzed by PCR. The expression levels of Wnt10a, LRP5/6, DKK1, and Periostin in cells stimulated with Periostin were analyzed by PCR. Wnt10a protein expression was analyzed by Western blotting and ELISA. Similar evaluations were performed with co-stimulation by Periostin and DKK1(Sample size:4). In each experiment, cells not stimulated with periostin served as the control group. Statistical analysis involved confirming the normal distribution of data using QQ plots, followed by one-way analysis of variance and post hoc Turkey’s test. Results: Migrating dental pulp stem cells expressed Wnt10a, and migration was additionally inhibited by its antagonist DKK1. Furthermore, Periostin stimulation increased Wnt10a secretion and suppressed DKK1. Conclusions: Periostin significantly increased Wnt10a expression and DPSC migration, while DKK1 inhibited these effects.

## 1. Introduction

The goal of pediatric dental treatment is to promote normal growth and development. Among these, the physiological root resorption of deciduous teeth and the physiological root formation and growth of immature permanent teeth play extremely significant role for oral development, and the pulp regulates these processes. Currently, pulp regeneration therapy has demonstrated safety and efficacy, emerging as a viable treatment option [1,2]. In pulp regeneration therapy, transplanted pulp stem cells secrete trophic factors that induce migration of surrounding stem/progenitor cells into the root canal [3]. It is hypothesized that the subsequently mobilized and proliferating cell population differentiates within the root canal microenvironment; however, many aspects remain unclear. Therefore, to enhance the therapeutic efficacy of pulp regeneration therapy, a deeper understanding of cell migration driven by trophic factors, which are central to the healing process, is considered necessary.

The Wingless-related integration site (Wnt) signaling pathway has been identified as a key signaling pathway involved in different stages of tooth development, such as cell fate determination, maturation, and differentiation, and as a pathway that regulates interactions throughout the organism’s lifetime [4,5,6,7]. Its regulation is achieved through the balance of ligand and agonist composition and distribution, occurring with spatiotemporal specificity. Regenerative responses are considered reconstructions of developmental processes, and attempts are being made to control stem cells and regenerate tissues by mimicking Wnt signaling [8,9,10]. Since Wnt10a is reported to be involved in tooth development and regeneration, refs. [11,12] understanding and reproducing its role in pulp regeneration is essential for realizing dental regenerative medicine.

Periostin is known as an extracellular matrix (ECM) protein present in lung, heart, skin, and periodontal ligament tissues, regulating collagen fiber formation [13,14]. Furthermore, its involvement in cell migration and survival has been reported in many fetal tissues, suggesting a contribution to developmental stages [15]. In tooth development, its expression at the epithelial-mesenchymal junction and involvement in controlling calcification have been reported [16,17]. More recently, its involvement in ECM cell interactions in vivo, such as promoting bone differentiation of bone marrow derived mesenchymal stem cells, has also been suggested [18]. Our analysis of previous dental pulp regeneration experiments has revealed that Periostin possesses ECM cell interactions with dental pulp stem cells, including the ability to promote proliferation and enhance the migration of type I collagen, the ECM component constituting dental pulp [19]. Activation of the Wnt signaling pathway by Periostin has been reported in osteoblasts, bone marrow stem cells, and vascular smooth muscle cells [20,21]. Periostin binds to integrins and activates various signaling pathways; among these, the Wnt signaling pathway is one activation pathway mediated via avβ3 [22,23]. Consequently, periostin can be considered a direct or indirect stimulator of Wnt. Effect of Postn may lead to inhibit excessive bone resorption and increase bone regenelation through the Wnt/β-catenin signaling pathways after ovariectomized (OVX) [24]. Consequently, from the perspective of cell migration, elucidating the relationship between Wnt10a and periostin in dental pulp stem cells is considered necessary for a deeper understanding of the mechanisms of dental pulp regeneration [10,11,12,19,20,21].

Therefore, this study hypothesizes that periostin stimulates Wnt10a, thereby promoting dental pulp regeneration by enhancing the migration of dental pulp stem cells. First, we analyzed the involvement of Wnt10a in cell migration during dental pulp regeneration. Next, we analyzed the changes occurring when DKK1, an antagonist of Wnt10a, was added to cells migrating under the influence of periostin. This allowed us to examine whether the Wnt signaling mediated by periostin could be a target for dental pulp regeneration therapy, specifically whether periostin could serve as a non-cellular dental pulp regeneration inducer.

## 2. Materials and Methods

### 2.1. Isolation of Human Dental Pulp Stem Cells

Dental pulp and periodontal ligament tissue recovered from deciduous teeth extracted from patients visiting Department of Pediatric Dentistry at Aichi Gakuin University Dental Hospital were isolated using an enzymatic digestion method based on the protocol of Sakatoku et al. [19]. First, the collected dental pulp and periodontal ligament were finely minced with the addition of 2 mg/mL collagenase (Wako, Osaka, Japan), then agitated at 37 °C for 30 min to disperse the tissue. Dulbecco’s Modified Eagle’s Medium (DMEM) was then added, followed by centrifugation at 4 °C at 1000 rpm for 5 min. After discarding the supernatant, DMEM was added again and the cells were seeded onto a dish. This established individual-derived deciduous tooth pulp stem cells (SHED) and periodontal ligament cells (PDL). SHED were cultured using fetal bovine serum (FBS) and DMEM at 37 °C in a 5% CO_2_ environment.

### 2.2. Preparation of Human Dental Pulp Stem Cell Conditioned Media

SHEDs passaged up to the third generation were cultured to 80% confluence, then switched to serum free medium. After 24 h, the conditioned media (CM) were collected. The collected supernatant was concentrated approximately 10-fold by centrifugation using a Vivaspin™ 6-3K (231700137, cytiva, Tokyo, Japan), and Protease Inhibitor (Halt™ Protease Inhibitor single use cocktail, Thermo Scientific, Rockford, IL, USA) was added. The protein concentration in CM was quantified using the Qubit^®^ Assays kit (Thermo Fisher Scientific, Tokyo, Japan).

Cell collection for this study was conducted with the approval of the Ethics Committee of the School of Dentistry, Aichi Gakuin University (Approval No. AGUD621).

### 2.3. Analysis of Regenerated Tissue in a Mouse Ectopic Tooth Root Transplant Model

(1) Ectopic Tooth Root Transplant Model

The ectopic tooth root transplant model was based on the method of Hayashi et al. and was used to evaluate the dynamics of migrating cells during pulp regeneration induced by CM.

Grafts were prepared by cutting human permanent teeth, extracted from patients visiting Department of Pediatric Dentistry at Aichi Gakuin University Dental Hospital, into 6 mm wide cylindrical sections, enlarging the root canal to 2 mm, completely removing all residual pulp, and sealing one end with hydraulic cement. The CM solution prepared in 2. was then mixed with collagen TE (Nitta Gelatin, Tokyo, Japan) to achieve a final concentration of 10 μg/mL and injected into the root canal to create the graft. These grafts were transplanted into the peritoneal cavity of 5–6-week-old male SCID mice (SLC Japan, Shizuoka, Japan). BrdU (150 μg/kg) was injected around the graft 3 days after transplantation. Transplantation was performed on 4 mice over 7 days. Seven days after transplantation, the grafts were recovered and immersed in 4% paraformaldehyde (Nakarai Tesque, Kyoto, Japan) at 4 °C overnight for fixation. Following this, decalcification was performed using Kalkitox (Wako, Osaka, Japan) at 4 °C for one week, after which 8 μm paraffin sections were prepared.

Animal experiments in this study were conducted in accordance with the Aichi Gakuin University School of Dentistry Animal Experimentation Regulations and were approved by the Animal Experimentation Ethics Committee (Approval Number. AGUD499).

(2) Observation of regenerated pulp-like tissue

To evaluate the tissue regenerated by ectopic root transplantation, hematoxylin and eosin (HE) staining was performed on paraffin sections taken 7 days post transplantation. The regenerated pulp-like tissue was then observed under brightfield illumination using an optical microscope (BX53, Olympus, Tokyo, Japan).

(3) Observation of Migrating Cells within the Regenerated Pulp-like Tissue

To confirm the localization of BrdU labeled migrating cells, paraffin sections taken 7 days post transplantation were reacted overnight at 4 °C with mouse anti BrdU (129964001, Roche Diagnostics, Mannheim, Germany, 1:100) as the primary antibody. Biotinylated goat anti mouse IgG (ab6789, Abcam, Cambridge, UK, 1:500) as the secondary antibody. The ABC kit (K-6100, Vector Laboratories, Burlingame, CA, USA) was used for processing, and color development was performed with DAB. Observations were made using brightfield illumination with an optical microscope BX53 (Olympus, Tokyo, Japan). Additionally, using the same primary antibody, color development was performed with goat anti mouse Alexa488 (ab150117, Abcam, Cambridge, UK, 1:200) as the secondary antibody. To confirm the localization of Wnt10a expressing cells within the regenerated pulp-like tissue, a rabbit anti Wnt10a antibody (NBP1-76916, Novus Biologicals/Funakoshi, Tokyo, Japan, 1:400) as the primary antibody. The sample was incubated overnight at 4 °C. For the secondary antibody, donkey anti rabbit Alexa594 (ab150076, Abcam, Cambridge, UK, 1:200) was used, and the sample was incubated at room temperature for 1 h to perform fluorescent immunohistochemical staining. Following staining with each antibody, nuclei were stained with Hoechst 33342 (H3570, Thermo Fisher Scientific, Waltham, MA, USA). Observations were made using an optical microscope BX53 (Olympus, Tokyo, Japan).

(4) BrdU and Wnt10a Positive Cell Rates

The number of antibody positive cells in photographed paraffin sections was counted using the image analysis software ImageJ (version 1.54r, National Institutes of Health, Bethesda, MD, USA) to measure the number of BrdU and Wnt10a positive cells. Similarly, cells stained with Hoechst 33342 were counted as the total number of cells. The positive cell rate was then calculated by dividing the number of positive cells by the total number of cells.

(5) Molecular Biological Analysis in Regenerated Tissue Genetic Analysis qPCR

Ectopic tooth root grafts were performed using the same method as described in Section 2.3 (1). On days 3 and 7 post transplantation, pulp-like tissue from within the grafts was harvested, immersed in RNAlater^TM^ Stabilization Solution (Thermo Fisher Scientific, Waltham, MA, USA), and stored at −80 °C.

The recovered tissue was then homogenized using a bench top bead mill, ShakeMan 3 (BMS, Tokyo, Japan), and RNA was isolated. RNA isolation was performed using the pressure method with the Quick Gene RNA cultured cell Kit S (Kurabo, Tokyo, Japan). The isolated total RNA was standardized to 1 μg/10 μL and converted to cDNA using the PrimeScript™ 1st Strand cDNA Synthesis Kit (Takara, Shiga, Japan). Subsequently, the gene expression of *β-actin*, *Wnt10a*, *DKK1*, *Periostin*, and *LRP5/6* in regenerated dental pulp-like tissue were analyzed using a high-speed real time PCR system (GF-Q150, Kurabo) with primers shown in Table 1. The results were normalized to β-actin.

There was no significant difference in β-actin expression levels among the samples used, and it was stably expressed. β-actin expression was observed in all samples used in this experiment, and it was therefore designated as the housekeeping gene.

First, 2 µL of the converted cDNA, 5 µL of the fluorescent reagent (1 Quick One step RT-PCR Master Mix, Kurabo), and 3 µL of the primer for analysis were added to prepare a total of 10 µL of reagent.

After confirming no air bubbles were present in the reagent, it was added to the Quick PCR Chip (Kurabo) and loaded into the high-speed real time PCR system. The relative expression level of the target mRNA was determined using the ΔΔCt method. This involved calculating the Ct value of the obtained sample relative to the Ct value of the housekeeping gene β-actin (ΔCt) and then calculating the relative expression level as a percentage based on the ΔCt value of the control sample.

For the positive control in the above experiment, normal dental pulp mRNA extracted from the central incisors of SCID mice from which the grafts were recovered was used.

### 2.4. Evaluation of Migrating Cells Induced by Periostin Stimulation

(1) Identification of Cell Types with Migration Promoting Ability

Migration assays using the Oris^TM^ Pro Cell Migration Assay Kit (Platypus Technologies, LLC, Madison, WI, USA) were performed on SHED, PDL, and HUVEC cells were subjected to a migration assay using the Oris Pro Cell Migration Assay Kit (Platypus Technologies, LLC). Specifically, 5000 cells were seeded into each well of a 96 well plate containing Bio Compatible Gel (BCG) in the center. The plates were incubated for 24 h. The time point when BCG dissolution was confirmed was designated as 0 h, and that area was defined as the detection area. After a further 24 h incubation, cells migrated into the detection area, and the closed area was calculated. The initial wound area divided by the closed area after 24 h was expressed as a percentage and designated as the closed area. Each experiment was repeated with four independent biological replicates (N = 4), and each measurement was performed in (n = 4) technical replicates

(2) Evaluation of Changes in Migrating Cell Numbers

First, SHED was fluorescently labeled using Cyto-ID (Enzo Life Sciences, Inc., Farmingdale, NY, USA). Next, a Vivant-cell pot (Nepa Gene Co., Ltd., Chiba, Japan) was used. The bottom was filled with DMEM containing Periostin (100 μg/mL), DKK1 (100 μg/mL) (Proteintech Group, Inc., Rosemont, IL, USA), or both. A membrane with 8 μm pores was placed on top. Five thousand labeled SHED molecules were seeded onto the membrane and incubated. After 24 h, the membrane was removed, and fluorescence was measured.

(3) Gene Expression Changes in Periostin Stimulated Cells

Cells stimulated for 24 h with Periostin (100 μg/mL), DKK1 (100 μg/mL), or both were harvested. RNA was immediately isolated using the same method as in and converted to cDNA. The cDNA was analyzed by high-speed real-time PCR system using primers for *β-actin*, *Wnt10a*, *DKK1*, *Periostin*, and *LRP5/6* shown in Table 2, and normalized to β-actin. q-PCR assays were performed with biological replicates (N = 4), each analyzed in (n = 4) technical replicates.

There was no significant difference in β-actin expression levels among the samples used, and it was stably expressed. β-actin expression was observed in all samples used in this experiment, and it was therefore designated as the housekeeping gene.

(4) Protein Expression Analysis in Periostin Stimulated Cells

➀ Western Blot

Cells stimulated for 24 h with Periostin (100 μg/mL), DKK1 (100 μg/mL), or both were harvested. Protein was extracted using PRO-PREP^TM^ Protein Extraction Solution (70810256, INB Intron Biotechnology, Seongnam, Gyeonggi, Republic of Korea) to extract proteins. Protein concentration was quantified using the Qubit Assays kit (Thermo Fisher Scientific, Tokyo, Japan). Twenty-five micrograms of extracted protein were electrophoresed using the Novex Bolt^TM^ Gel Electrophoresis System (Thermo Fisher Scientific) (200 V, 22 min) and transferred using the iBlot2 Dry Blotting System (Thermo Fisher Scientific). After transfer, the iBind Flex Western System (Thermo Fisher Scientific) was used. The primary antibody was anti Wnt10a antibody (NBP1-76916, Novus Biologicals/Funakoshi, Tokyo, Japan, 1:1000 dilution), and the secondary antibody was anti Rabbit IgG antibody (abcam, Cambridge, UK, ab6720, 1:5000 dilution). Detection was performed using the ChemiDoc^TM^ Touch MP Imaging System (Bio Rad Laboratories, Hercules, CA, USA).

➁ ELISA

Proteins recovered as in ➀ were adjusted to 10 μg. These were used to quantify Wnt10a expression levels using an ELISA kit (ATXK26163, My Biosource, San Diego, CA, USA). All ELISA assays were conducted with (N = 4) independent biological samples, each measured in (n = 4) technical replicates.

### 2.5. Statistical Analysis

All data represent the mean ± standard deviation of (N = 4) independent biological replicates, each derived from the average of (n = 4) technical replicates unless otherwise stated. After creating a QQ plots and confirming the normal distribution, we performed the hypothesis test. Statistical analysis was performed using one way analysis of variance (ANOVA) and multiple comparison tests (Tukey’s method). Statistical significance was determined at a 5% significance level. Statistical analysis was performed using SPSS ver21.0^®^ (IBM, Armonk, NY, USA).

## 3. Results

### 3.1. Evaluation of Migrated Dental Pulp Stem Cells

**Figure 1:**
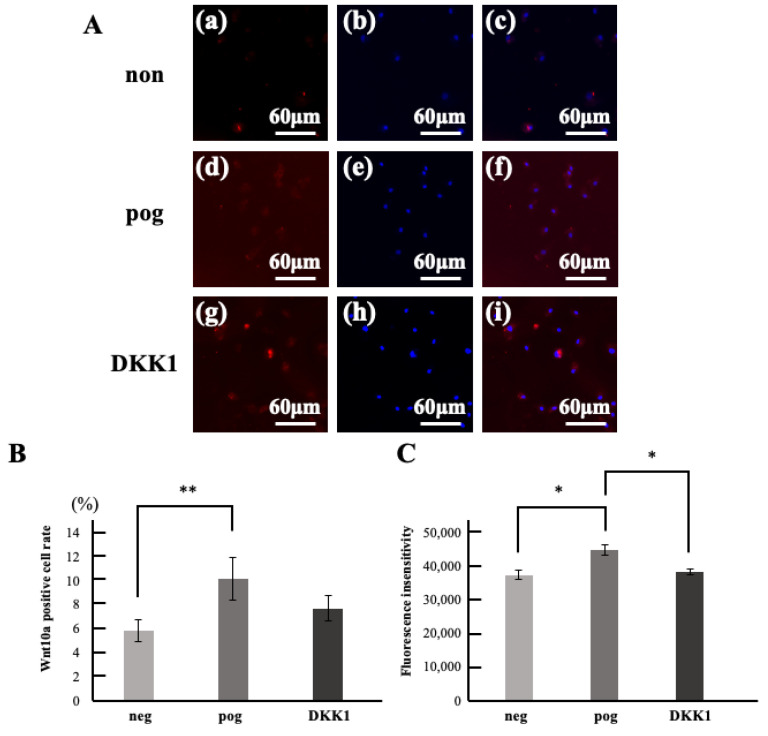
Wnt10a expression rate in migrated cells. (**A**) Double stained images of migrated cells with Wnt10a immunostaining (red) and hoechst33342 immunostaining (blue). (**a**–**c**) negative control; neg. (**d**–**f**) positive control; pog(FBS). (**g**–**i**) DKK1. (**a**,**d**,**g**) Wnt10a immunostained images. (**b**,**e**,**h**) hoechst33342 immunostained image. (**c**,**f**,**i**) merged images. (**B**) Wnt10a positive cell rate of migrated cells. (**C**) Fluorescence insensitivity. * *p* < 0.05, ** *p* < 0.01. All data represent mean ± standard deviation (n = 4).

Immunohistochemical staining of migrated dental pulp stem cells with Wnt10a showed a positivity rate of approximately 5% in the unstimulated group. The Wnt10a positive rate in the stimulated group was approximately 10%, representing a significant increase (Figure 1A,B). Furthermore, stimulation with DKK1, an antagonist of Wnt10a, reduced the Wnt10a positivity rate in migrating cells, eliminating the significant difference compared to the unstimulated group (Figure 1C).

### 3.2. Evaluation of Migrating Cells Within Regenerated Dental Pulp Tissue

**Figure 2:**
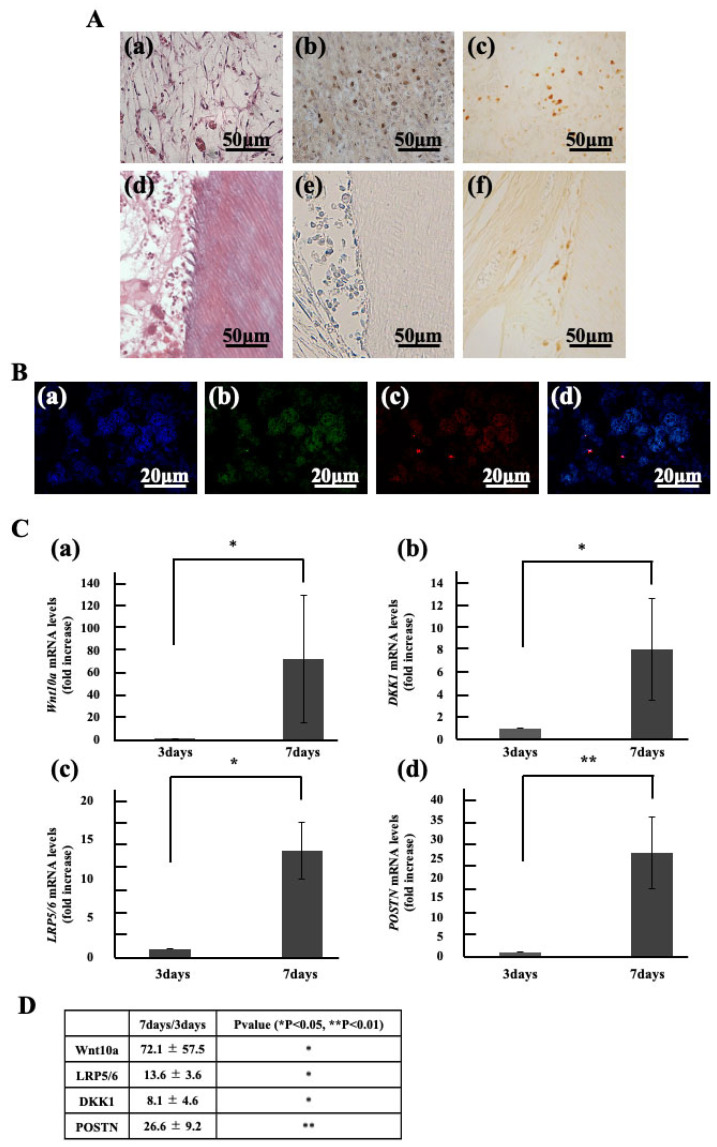
Localization and Gene Expression of Migrated Cells in Regenerated Dental Pulp. (**A**) HE stained image (**a**,**d**). Immunostained images of Regenerated pulp with BrdU (**b**,**e**), Wnt10a (**c**,**f**). (**B**) Double stained images of regenerated pulp-like tissue with Hoechst 33342 immunostaining (blue), BrdU immunostaining (green), and Wnt10a immunostaining (red). (**a**) Hoechst 33342 immunostaining. (**b**) BrdU immunostaining. (**c**) Wnt10a immunostaining. (**d**) merged images of Hoechst 33342, BrdU and Wnt10a. (**C**) Gene Expression in dental pulp. (**a**) Wnt10a, (**b**) DKK1, (**c**) LRP5/6, (**d**) Periostin (POSTN). (**D**) Foldchange values and statistical significance. * *p* < 0.05, ** *p* < 0.01. Relative quantification by ΔΔCt method after q-PCR analysis. All data represent mean ± standard deviation (n = 4).

In the ectopic root graft model, the group transplanted with culture supernatant showed regenerated dental pulp tissue rich in blood vessels and fibers within the root canal by day 7 (Figure 2 A(a,d)). However, no regenerated dental pulp tissue was observed in the group transplanted with Periostin or collagen only. Therefore, the regenerated dental pulp tissue from the supernatant transplant was analyzed. Cell from host tissue labeled were observed within the regenerated dental pulp tissue (Figure 2A(b)). These BrdU positive cells were detected at the cellular influx sites within the root and around blood vessels, but not around the root canal walls (Figure 2A(e)). In contrast, Wnt10a positive cells were detected not only at the cellular influx sites and around blood vessels but also around the root canal walls (Figure 2A(c,f)). Next, double fluorescent immunohistochemical staining was performed, and the positivity rates for BrdU and Wnt10a were calculated. Within the regenerated dentin-pulp complex, 22% BrdU positive cells and 11% Wnt10a positive cells were observed. Furthermore, among the BrdU positive cells, 47% co-expressed Wnt10a (Figure 2B). Subsequently, tissue was collected from the root canal immediately after transplantation and again on day 7 post transplantation. Gene expression was analyzed and compared using qPCR. The results showed a significant increase in expression: approximately 7-fold for *Wnt10a*, approximately 8-fold for DKK1 (an antagonist of Wnt10a), and approximately 13-fold for the receptor *LRP5/6*. Periostin expression also significantly increased approximately 26-fold (Figure 2C).

### 3.3. Evaluation of Migrating Cells Following Periostin Stimulation

(1) Identification of Cell Types Exhibiting Enhanced Migration

**Figure 3:**
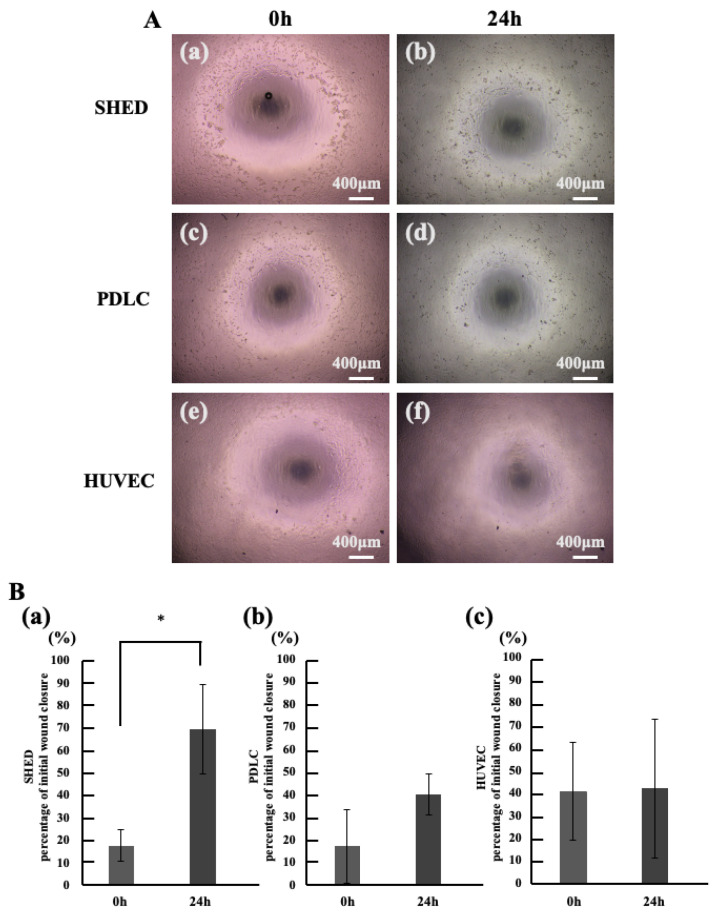
Investigation of Cell Types Promoting Migration Induced by Periostin Stimulation. (**A**) Microscopic image of the Cell Migration Assay. (**a**,**b**) SHED. (**c**,**d**) PDLC. (**e**,**f**) HUVEC. (**a**,**c**,**d**) 0 h, (**b**,**d**,**f**) 24 h. (**B**) percentage of initial wound closure in Migration Assay. (**a**) SHED, (**b**) PDLC, (**c**) HUVEC. * *p* < 0.05. All data represent mean ± standard deviation (n = 4).

Periostin stimulation was applied to dental pulp stem cells, periodontal ligament cells, and vascular progenitor cells all expected to migrate in vital pulp therapy followed by scratch assays (Figure 3A). The results showed a significant increase in percentage of initial wound closure for dental pulp stem cells (Figure 3B(a)). However, no significant difference in closed area was observed in periodontal ligament cells or vascular progenitor cells (Figure 3B(b,c)).

**Figure 4:**
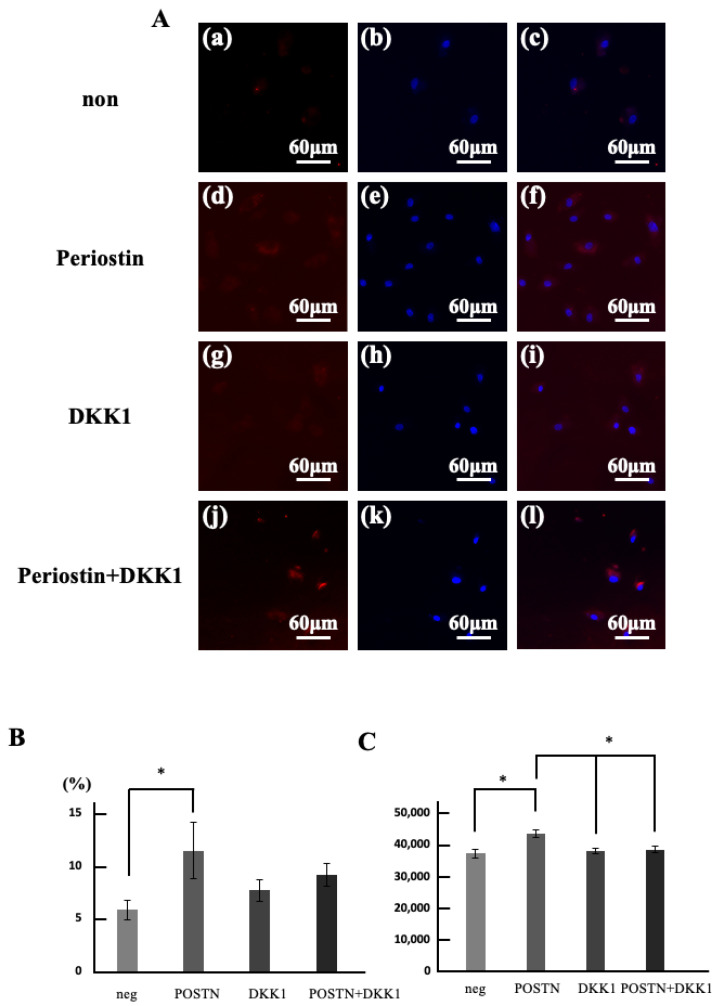
Changes in the number of migrated cells and the rate of Wnt10a-positive cells induced by periostin stimulation. (**A**) Double stained images of regenerated pulp-like tissue with Wnt10a immunostaining (red) and hoechst33342 immunostaining (blue). (**a**–**c**) neg. (**d**–**f**) POSTN. (**g**–**i**) DKK1. (**j**–**l**) DKK1 and POSTN. (**a**,**d**,**g**,**j**) Wnt10a immunostained images. (**b**,**e**,**h**,**k**) Hoechst33342 immunostained image. (**c**,**f**,**i**,**l**) superimposed images of Wnt10a and hoechst33342. (**B**) Double stained images of regenerated pulp-like tissue with Wnt10a immunostaining (red) and Lectin immunostaining (green). (**a**–**d**) Wnt10a immunostained images. (**e**–**h**) Lectin immunostained image. (**i**–**l**) superimposed images of Wnt10a and Lectin. (**a**,**e**,**i**) 3 days. (**b**,**f**,**j**) 7 days. (**c**,**g**,**k**) 14 days. (**d**,**h**,**l**) 21 days. (**C**) Area of angiogenesis within the regenerated pulp-like tissue. * *p* < 0.05. Relative quantification by ΔΔCt method after q-PCR analysis. All data represent mean ± standard deviation (n = 4).

(2) To verify whether periostin stimulation activates Wnt10a in dental pulp stem cells that showed significant differences in Section 3.1, we first measured changes in the Wnt10a-positive rate of migrating cells induced by periostin (Figure 4A). The group stimulated with Periostin showed a significantly increased number of migrating cells compared to the unstimulated group (Figure 4B). Furthermore, the cell migration counts in the group stimulated only with DKK1 showed no change compared to the unstimulated group but was significantly reduced compared to the Periostin stimulated group. Moreover, the cell migration counts in the group stimulated simultaneously with Periostin and DKK1 was significantly reduced compared to the Periostin stimulated group and showed no significant difference compared to the unstimulated group. The Wnt10a positive rate significantly increased with Periostin stimulation (Figure 4C).

(3) Gene changes in Periostin stimulated cells

**Figure 5:**
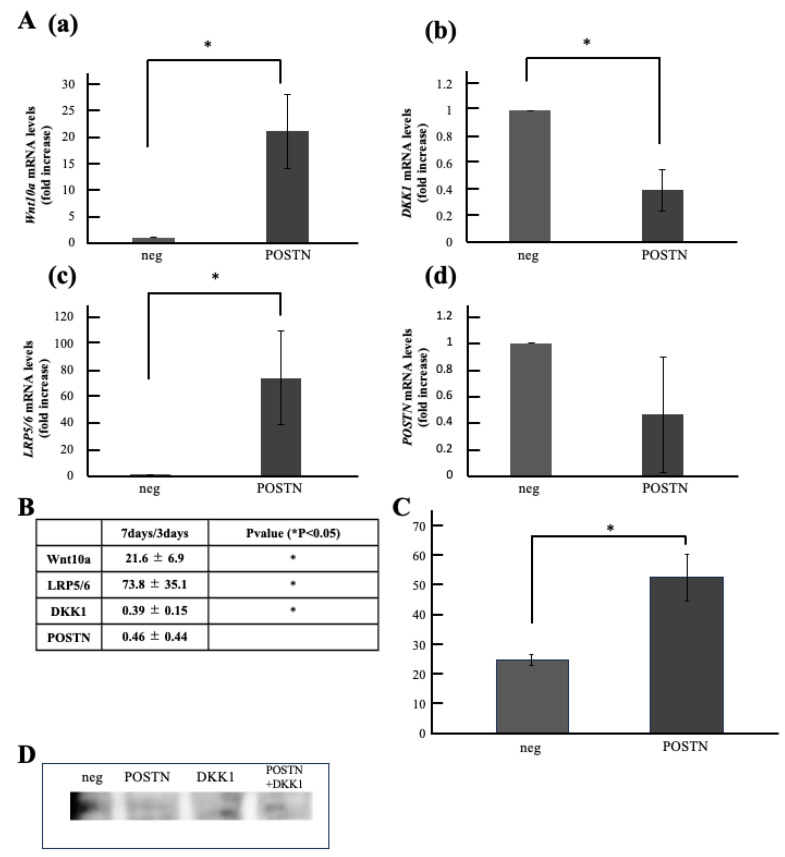
Comparison of Wnt10a Expression Levels in Periostin-Stimulated Cells. (**A**) Gene Expression in Periostin-Stimulated Cells. (**a**) Wnt10a, (**b**) DKK1, (**c**) LRP5/6, (**d**) Periostin(POSTN). (**B**) Foldchange values and statistical significance. (**C**) Wnt10a Expression in Periostin-Stimulated Cells by ELISA. (**D**) Wnt10a Expression in Periostin-Stimulated Cells by Western blotting. * *p* < 0.05. Relative quantification by ΔΔCt method after q-PCR analysis. All data represent mean ± standard deviation (n = 4).

Next, we analyzed the unstimulated group and the Periostin stimulated group by qPCR and compared the changes in Wnt related genes induced by Periostin stimulation. The results showed that gene expression levels in the Periostin stimulated group were significantly increased, approximately 21-fold for *Wnt10a* and approximately 73-fold for the receptor *LRP5/6*. Conversely, *DKK1* expression was significantly reduced to 0.4-fold. *Periostin* expression levels remained unchanged (Figure 5A).

(4) Comparison of Wnt10a Expression Levels in Periostin Stimulated Cells

Wnt10a expression levels were compared using ELISA. The results showed that Wnt10a expression was significantly increased in the Periostin stimulated group compared to the unstimulated group (Figure 5B). Furthermore, Wnt10a expression was confirmed in all groups by Western Blotting (Figure 5C).

## 4. Discussion

Cell migration is a fundamental trophic effect underpinning the development, homeostasis maintenance, and repair/regeneration of biological tissues. Mesenchymal stem cells (MSCs), which act as reservoirs of trophic factors, play a pivotal role in regenerative medicine by migrating to and adhering to damaged tissues [25,26,27]. However, the efficiency with which MSCs reach target tissues after systemic administration is not high [27]. The migration process is influenced by both the chemical microenvironment, composed of various trophic factors, and the mechanical microenvironment, composed of cells and the extracellular matrix (ECM) [28,29,30]. Consequently, extensive research has focused on controlling this process, including identifying migration promoting factors, introducing genes involved in migration, developing scaffold materials, or combining these approaches [30,31]. While the therapeutic efficacy of stem cell applications is now well established, their use in dentistry, an area with many acute treatment needs, remains challenging due to issues like amplification, storage duration, immune rejection, and cost [25]. Consequently, the development of storable, non-cellular dental regenerative agents that do not require stem cells is an urgent priority [19].

The Wnt signaling pathway is one of the key pathways governing cell migration, and Wnt10a, in particular, is deeply involved in tooth development and regeneration [11,12]. Thus, we first analyzed whether Wnt10a is involved in migrating dental pulp stem cells and further examined the localization of Wnt10a and migrating cells distributed in regenerated dental pulp using both in vitro and in vivo approaches. In vivo, approximately 10% of migrating dental pulp stem cells were Wnt10a positive, showing a significant increase compared to cells not subjected to migration stimulation. Within the regenerated pulp-like tissue formed by ectopic root transplantation, BrdU labeled cells represent those that migrated from surrounding tissues into the root canal. The mechanism of cell migration involves a sequential series of events occurring around blood vessels and on endothelial cells: tethering and rolling, activation, arrest, transmigration or diapedesis, and migration [28,29,32]. Therefore, BrdU positive cells were likely localized at the cell influx sites and around blood vessels. Wnt signaling is one pathway deeply involved in cell migration, reported to promote the migration of mesenchymal stem cells and progenitor cells during vascular, neural, and bone differentiation [33,34,35]. Since root growth ceases with pulp necrosis, pulp preservation is critically important in pediatric dental treatment [36,37,38,39,40]. Thus, vital pulp therapy, including pulp capping and pulpotomy, is performed with the aim of minimizing the impact on growth and development by preserving or partially recreating the physiological state as much as possible [36,37,38]. The most standard method for pulp capping or pulpotomy is the calcium hydroxide technique. It has been reported that Wnt signaling is also involved in the migration of pulp stem cells during this process. Furthermore, Wnt3a and Wnt10a are not normally expressed within the pulp, but their expression has been reported in the pulp following pulp capping with MTA [12,41], indicating that migrating cells express these molecules. Similarly, in the regenerated pulp from this experiment, *Wnt10a* expression levels increased sharply, and Wnt10a was co-expressed in 47% of migrating cells. Consequently, it is considered that cells migrating during the regenerative response express Wnt10a. Furthermore, it is known that biological phenomena mediated by Wnt signaling are precisely regulated by agonists and antagonists [7,10]. Multiple cell types and trophic effects are involved in dental pulp regeneration. This likely explains the simultaneous increase in DKK1. Furthermore, in isolated dental pulp stem cells, approximately 11% of migrating cells were Wnt10a positive. Furthermore, in the presence of the antagonist DKK1, both the number of migrating cells and the Wnt10a positivity rate decreased. This suggests that Wnt10a plays a role in governing the migration of dental pulp stem cells not only during pulp development and repair but also during the regenerative response. DKK1 inhibits not only Wnt10a but also canonical Wnt signaling molecules such as Wnt3a. Wnt3a, like Wnt10a, has been reported to be involved in dentin differentiation, and its co-transplantation with hydroxyapatite after Vital Pulp Therapy (VPT) has been shown to promote dentin differentiation [42]. This experiment did not extend to Wnt10a knockout. Future plans include conducting more detailed migration experiments using Wnt3a and Wnt10a knockout cells to elucidate the migration mechanisms involved in dental pulp regeneration. Dentin phosphoprotein (DPP), one of the dentin matrix proteins, promotes interactions between Wnt5a and Frizzled 5 and LRP6, inducing the nuclear translocation of β-catenin [43]. As a result, it has been reported to promote dentin differentiation in stem cells. In other words, while Wnt3a, 5a, and 10a have all been reported to be involved in dentin differentiation through activation of the β-catenin pathway, only Wnt10a has been reported to promote proliferation and migration of dental pulp stem cells.

Periostin is known as an ECM protein that maintains tissue structure by binding to other ECM components such as collagen I, fibronectin, and tenascin C [11,18]. Simultaneously, it has been revealed to function as a matricellular protein that influences cell migration by mediating interactions between cells and the substrate.

Periostin binds to integrins, cell surface receptors, and transmits signals into the cell; one such pathway is Wnt signaling [39,40,41,42]. This experiment also demonstrated that periostin strongly promotes migration in deciduous tooth pulp stem cells. Gene analysis of periostin-stimulated pulp stem cells showed increased wnt10a expression and decreased DKK1 expression. Furthermore, ELISA revealed increased wnt10a protein expression, which decreased upon co-stimulation with DKK1. Additionally, ELISA confirmed increased Wnt10a protein expression. Furthermore, the migration promoting effect on dental pulp stem cells was attenuated by DKK1, and immunohistochemical staining showed suppressed Wnt10a positivity. So, it was clarified that Periostin stimulated migration of dental pulp stem cells is mediated by Wnt10a induction. In this study, *Periostin* expression levels remained unchanged following Periostin stimulation of dental pulp stem cells (i.e., in vitro stimulation) but showed altered expression levels within regenerated dental pulp. Periostin has been reported to possess a multi domain structure and act as a scaffold for assembling interacting proteins. Our previous results also showed that in regenerated pulp, Periostin was highly expressed in the pulp matrix on day 7, when cell migration was thought to be active, and faded by day 21, when sufficient pulp regeneration was observed. Therefore, it was suggested that promoting the migration of periostin in dental pulp regeneration may require its three-dimensional arrangement as a scaffold. This study did not perform functional inhibition experiments of periostin or Wnt10a, nor did it evaluate Wnt downstream signaling. Performing these could clarify the relationship between periostin and Wnt10a.

When pulp regeneration therapy or vital pulp therapy is applied, migration occurs during the early healing phase. This indicates that migration promotion is one essential property that non-cellular dental regeneration inducers should possess. The migration promoting effect of Periostin was observed in pulp cells but not in periodontal ligament cells or vascular progenitor cells. This suggests that Periostin is effective for vital pulp therapy. Furthermore, the migration promoting effect of Periostin was also observed in the regenerated pulp matrix. but not on periodontal ligament cells or vascular progenitor cells. This suggests Periostin is effective for vital pulp therapy, which preserves the vital pulp. Furthermore, periostin may possess migration-promoting activity via Wnt signaling, similar to hydroxyapatite preparations and MTA currently used clinically.

## 5. Conclusions

In this study, Periostin significantly increased Wnt10a expression and DPSC migration, while DKK1 inhibited these effects.

## Figures and Tables

**Table 1:** Mouse primers for reverse transcription-polymerase chain reaction.

Gene Name		5′←DNA Sequence→3′	Amplicon Size	Accession Number
β-actin	Forward	CATTGCTGACAGGATGCAGAAGG	138	NM_007393
	Reverse	TGCTGGAAGGTGGACAGTGAGG		
Wnt10a	Forward	GCTCCTGTTCTTCCTACTGCTG	119	NM_009518
	Reverse	ATGTCAGGCACACTGTGTTGGC		
DKK1	Forward	ATCTGTCTGGCTTGCCGAAAGC	115	NM_010051
	Reverse	GAGGAAAATGGCTGTGGTCAGAG		
Periostin	Forward	AGACGACCTTTCATCATTTAGAGCA	87	MN_001198765
	Reverse	GCAAAGAGCGTGAAGTGACCAT		
LRP5/6	Forward	CCTCACCATTGATTATGCCGACC	110	NM_008513
	Reverse	GATCGTCAGCTATCACCATGCG		

**Table 2:** Human primers for reverse transcription-polymerase chain reaction.

Gene Name		5′←DNA Sequence→3′	Amplicon Size	Accession Number
β-actin	Forward	CACCATTGGCAATGAGCGGTTC	135	NM_001101
	Reverse	AGGTCTTTGCGGATGTCCACGT		
Wnt10a	Forward	GTGCTCCTGTTCTTCCTACTGC	128	NM_025216
	Reverse	CCTGGCAATGTTAGGCACACTG		
DKK1	Forward	GGTATTCCAGAAGAACCACCTTG	125	NM_012242
	Reverse	CTTGGACCAGAAGTGTCTAGCAC		
Periostin	Forward	CAAGGGAGAAACGGTGCGATT	114	NM_014208.3
	Reverse	AAGTAGGCTGAGGAAGGTGCTAA		
LRP5/6	Forward	GGACACCAACATGATCGAGTCG	141	NM_002335
	Reverse	CGCTCAATGCTGTGCAGATTCC		

## Data Availability

All data generated or analyzed during this study are included in this published article.

## Abbreviations

The following abbreviations are used in this manuscript:

DPSC	Dental pulp stem cell
SHED	Stem cells from human exfoliated deciduous teeth
WNT	Wingless-related integration site
LRP	Low-density lipoprotein receptor-related protein
DKK	Dickkopf related protein
CM	Conditioned medium
BrdU	Bromodeoxyuridine
